# Gait alteration during turning while walking in older adults with benign paroxysmal positioning vertigo

**DOI:** 10.1080/07853890.2024.2402952

**Published:** 2024-11-16

**Authors:** Haziqah Nasruddin, Maria Justine, Alia Alghwiri, Haidzir Manaf

**Affiliations:** a Physiotherapy Unit, Hospital Tuanku Jaafar, Negeri Sembilan, Malaysia; b Centre for Physiotherapy Studies, Faculty of Health Sciences, Universiti Teknologi MARA, Puncak Alam, Selangor, Malaysia; c Department of Physiotherapy, School of Rehabilitation Sciences, The University of Jordan, Amman, Jordan

**Keywords:** Gait, older adults, spatiotemporal parameters, turning, vertigo, walking

## Abstract

**Background:**

Older adults with Benign Paroxysmal Positioning Vertigo (BPPV) may present with unsteadiness that affects gait patterns.

**Objective:**

This study investigated the spatiotemporal gait parameters and indicators of turning difficulty during the Timed Up and Go (TUG) test in older adults with BPPV.

**Methods:**

This case-controlled study collected data from older adults aged 65 and above with BPPV, young adults with BPPV and older adults without BPPV. Postural stability and self-perception of stability were measured using the Functional Gait Analysis and the Malay version of the Dizziness Handicap Inventory, respectively. The spatiotemporal gait parameters were recorded using a camera. The one-way ANOVA test was used for statistical analysis.

**Results:**

Older adults with BPPV presented with alteration in gait parameters (time and number of steps) compared to older adults without BPPV and adults with BPPV during the TUG test (*p* < 0.05). During the straight walking tasks of the TUG test, there were significant differences in stride length and velocity between the three groups (*p* < 0.05). Older adults with BPPV presented with less pivoting and took longer time and more steps to complete the turning phase of the TUG.

**Conclusions:**

The findings suggest that gait performance was compromised in older adults with BPPV.

## KEY MESSAGES

1. During the straight walking portion of the TUG test, older adults with BPPV showed significant reductions in stride length and walking velocity when compared to the other two groups, suggesting a greater impact of BPPV on gait in this population.
2. In the turning phase of the TUG test, older adults with BPPV required more time, took more steps, and demonstrated less efficient pivoting compared to older adults without BPPV and younger adults with BPPV, indicating increased difficulty in balance and maneuverability.

## Introduction

Benign paroxysmal positional vertigo (BPPV) is the most common peripheral vestibular disorder that affects one-third of older adults [1]. The pathophysiology of BPPV involves the semicircular canals becoming sensitive to gravity, which can lead to abnormal stimulation of the semicircular canals, resulting in vertigo symptoms. Individuals with BPPV may experience dizziness, vertigo, imbalances, and unsteadiness while walking, including impaired gait performance [2, 3]. Consequently, these individuals, particularly older adults, may have difficulties with postural control, especially during challenging motor tasks such as turning or changing direction while walking. A large retrospective study of BPPV patients seen over five years reported a high prevalence of self-reported falls (47.7%), and more than half of the patients (54%) presented with abnormal postural stability [4]. Older adults are more vulnerable to the consequences of BPPV due to age-related factors such as cognitive decline, and frailty. Therefore, an investigation is needed to explore how BPPV affects older adults’ gait characteristics during challenging movements such as turning.

Turning is a complex task that incorporates changes in spatial-temporal parameters and coordination of axial segments [5–7]. A turn is initiated by rotation of the head, followed by the trunk, pelvis, and feet, which move toward the inner side of the turn. The vestibular system contributes to postural stability and visual stabilization through the vestibulospinal and vestibulo-ocular reflexes [8]. Dysfunction of the semicircular canals, made up of the kinetic labyrinth that senses angular acceleration or rotation of the head, can disrupt postural control. Hence, alteration of balance while turning in adults with BPPV may cause alteration of spatiotemporal gait characteristics, such as taking a long time during turning to maintain their postural stability. A previous study showed that the spatial-temporal characteristic of older adults during turning demonstrates shorter step length, longer step time, and slower step speed than young adults [9]. Furthermore, there are four indicators of turning difficulty among older adults, namely, staggering during the turn, absence of pivoting during the turn, using five or more steps or weight shifts, and taking 3 s or longer to accomplish the turn [10]. Therefore, older adults with BPPV symptoms such as vertigo, imbalance, and nausea often struggle to perform turning tasks safely and are at high risk of falling.

The Timed Up and Go (TUG) test is a useful measurement tool for physical mobility, which can be used either as a screening test or as a descriptive tool. The TUG test measures the time taken for an individual to stand up from a chair, walk 3 meters, turn 180°, walk 3 meters back to the chair, and sit down [11]. The TUG has been used to measure mobility and dynamic balance in various conditions, such as stroke survivors, multiple sclerosis, Parkinson’s disease, and older adults [9, 10, 12–15]. Consequently, no study has examined gait parameters and indicators of turning difficulty while walking among older adults with BPPV. The mobility tool using the TUG test with a video recording may be a useful and simple measurement that can be used to assess gait parameters among older adults with BPPV.

In order to have a better understanding of the turning deficits in older adults with BPPV, this study aims to determine the spatio-temporal gait parameters and indicators of turning difficulty during the TUG test in older adults with BPPV compared to older and young adults without BPPV. We hypothesized that older adults with BPPV would take more steps and longer time to complete the TUG test than other groups, especially during turning tasks.

## Methods

### Participants

Twenty-two older adults with BPPV, 22 younger adults with BPPV and 22 older adults without BPPV participated in this case-control study. A power analysis based on the previous study, with an estimated sample of 66 participants, would provide 80% power with a risk of type I error of 0.05 [16],. Participants were recruited from a government hospital through purposive sampling. The inclusion criteria were as follows: (1) BPPV diagnosed by the otorhinolaryngology specialist through positional testing, which involves provoking vertigo and observing nystagmus characteristic of BPPV such as Dix-Hallpike (posterior canal BPPV) and supine roll (lateral canal BPPV), depending on the involved canal, (2) older than 65 years old for older adults with and without the BPPV group, (3) age range between 40 and 64 years old for adults with BPPV group, (4) able to read and understand Malay or English Language, and (5) able to follow one-step commands. Participants were excluded if they had an acute vertigo symptom and other neurological and orthopaedic conditions that can affect balance performance (e.g. peripheral neuropathy, cerebrovascular disease, paresis, joint deformities, osteoarthritis, and rheumatoid arthritis). Before data collection, participants signed an informed consent form approved by the institutional ethics committee. The ethics approval was obtained from National Medical Research Register (NMRR-18-3881-40816) and Research Ethics Committee of Universiti Teknologi MARA (REC/358/19).

### Testing procedure

Participants underwent the testing procedure within one week of the onset of vertigo complaints. The demographic data were recorded after signing the informed consent form. Perception of stability of the participants was measured using the Dizziness Handicap Inventory (DHI) and postural stability using Functional Gait Analysis (FGA). All outcomes were measured by the same physiotherapist with ten years of experience in vestibular rehabilitation.

The DHI is a 25-item tool that rates the participant’s self-perception of disability from dizziness. The items were divided into a physical subscale (28 points), an emotional subscale (36 points), and a functional subscale (36 points). Each response is scored as 4 points (yes), 2 points (sometimes), and 0 points (no), and the total score ranges from 0 (no perceived disability) to 100 (maximum perceived disability) [17]. Other researchers have classified DHI scores into mild handicap (0–30), moderate handicap (31–60), and severe handicap (61–100) [18]. In this current study, the Malay version of DHI (MyDHI) was used in this current study. MyDHI showed acceptable internal consistency based on an alpha (α) value of 0.74. The Cronbach’s Alpha coefficient for MyDHI was acceptable, and the score was comparable to the original English version of DHI (α = 0.89) [19].

The FGA outcome measure is a 10-item tool which evaluates postural stability during various walking tasks [20]. The total FGA score is 30. A score <22 on FGA indicates that individuals with vestibular disorder were six times more likely to increase the risk based on the TUG test score. The FGA has excellent inter-rater reliability (ICC= 0.84) and excellent intra-rater reliability (ICC= 0.83) for patients with vestibular disorder [21]. The FGA also has concurrent validity with DHI (*r*= −0.64), a number of falls in the previous four weeks (*r*= −0.66), and adequate validity with the TUG test (*r*= −0.50) for patients with the vestibular disorder [21].

After clinical assessment, participants were asked to perform the TUG test. The TUG test measures the time taken for an individual to stand up from a chair, walk 3 meters, turn 180°, walk 3 meters back to the chair, and sit down [11]. Several researchers have used the TUG test to investigate gait parameters among the older adults population [10, 22] and adults with BPPV [23]. The normative value for the TUG shows that healthy older adults aged 60 to 69 years score 9.0 s, 70 to 79 years old score 10.2 and individuals 80 to 99 years old score 12.7 [24].

Before testing, the researcher explained the TUG test procedure to the participants. Participants were not allowed to wear any footwear during the trial. The participants conducted three trials before the actual test. First, the participants were seated in a chair with a 43 cm seat height of 20 cm armrest height. They were correctly seated with hips to the back of the seat. Next, the researcher instructed ‘GO’ and the participant stood up, walked 3 meters to the marker on the floor, turned around, walked back to the chair and sat down at a regular pace. Two stationary video cameras were mounted on a tripod and placed 6 meters away on the sagittal plane of the movement to record spatiotemporal gait parameters (type of turn, turn time, turn steps, stride time, stride length, and stride velocity) and 3 meters away on the frontal plane of movement to record the gait performance (total number of steps, total time taken) during TUG. Participants performed the TUG test without using walking aids. Performing the TUG test without the use of walking aids is essential to assessing an individual’s true functional mobility and balance abilities without external support.

All data were analyzed using Kinovea software. Kinovea is a free 2D motion analysis software widely used among researchers in gait analysis [25, 26]. We obtain our primary outcomes: time and number of steps taken to complete the TUG test. We further divided the TUG test into turning and straight walking phases to gain information about each phase. A turn is defined as the beginning and end of the 180-degree reversal of direction at the turn line on the floor while walking. The last heel strike before initiation of the reversal of direction is designed to be the beginning of the turn, while the heel strike of the first step progressing in a direct line back to the chair is the end of the turn [10]. The following results were obtained from a video recording: the number of steps and the time taken during the turning phase, the stride length, stride time and stride velocity during the straight walking phase.

The indicator of turning difficulty analysis was adopted from the previous study [10]. To maintain the reliability of the recorded video analysis, two experienced physiotherapists (>10 years of clinical experience) were appointed as research assistants. The principal investigator explained to the research assistants the procedure of video analysis. The sample data of the recorded video were randomized, and analysis was performed by the two researcher assistants. Any disagreement on the data found was consulted and discussed with the principal investigator.

Data analysis was performed using IBM SPSS statistical software version 21 (IBM SPSS, Armonk, NY). Data were screened for error and missing data and checked for normal distribution with the statistical normality test, skewness, and kurtosis. The mean, standard deviation, and range were calculated for each variable and data were presented in the table and line graph. A descriptive analysis of demographic characteristics was performed. A one-way ANOVA test was used to compare the spatiotemporal gait parameters among the three groups. The statistical significance was established at *p* < 0.05. The turning characteristics were described in frequency and percentage.

## Results

### Demographic data

This study included 66 participants after screening 28 older adults with BPPV, 25 adults with BPPV and 22 older adults without BPPV over a period of 3 months. The characteristics of the participants are shown in Table 1. There were no significant differences in height, weight and BMI in the three groups (*p* > 0.05) and no significant differences in duration of illness (*p* = 0.172) and DHI (*p* = 0.162) between older adults with BPPV and adults with BPPV. There was a significant difference in FGA among the three groups, with older adults with BPPV showing the lowest mean (*p* = 0.001), indicating poorer gait function.

**Table 1.** Characteristics of the participants.

Variable	OAWBPPV (*n* = 22)	OABPPV (*n* = 22)	ABPPV (*n* = 22)	*P*-value
	Means ± SD			
Age	69.9 ± 4.7	70.4 ± 5.4	55 ± 7.3	0.001*
Height	158.1 ± 7.4	154.9 ± 6.1	159.7 ± 5.9	0.052
Weight	62.1 ± 12.8	64.4 ± 12.5	65.7 ± 10.8	0.608
BMI	24.6 ± 4.7	26.4 ± 5.2	25.4 ± 3.4	0.406
Duration of illness		9.8 ± 6.6	8.1 ± 5.6	0.381
Dizziness Handicap Inventory		38.9 ± 16.9	47.3 ± 19.1	0.129
Functional gait assessment	27.5 ± 2.2	19.5 ± 6.6	23.9 ± 4.9	0.001*

* *p*<.05; One-way ANOVA,, BPPV: Benign Paroxysmal Positioning Vertigo, OAWBPPV: older adults without BPPV, OABPPV: older adults with BPPV, ABPPV: adults with BPPV.

### Total time and number of step TUG test

Table 2 shows the gait parameters between the three groups. There was a significant difference in the time taken to complete the TUG test among the three groups: F(2, 63) = 11.03, *p* < 0.01. Post hoc comparisons revealed that older adults with BPPV took a significantly longer time to complete the TUG test than older adults without BPPV and adults with BPPV (*p* = 0.001, respectively), while, between the adults with BPPV and older adults without BPPV, no significant differences were found.

**Table 2.** Gait parameters between the three groups.

Variable	OAWBPPV (*n* = 22)	OABPPV (*n* = 22)	ABPPV (*n* = 22)	*P*-value	OAWBPPV – OABPPV	OAWBPPV – ABPPV	OABPPV – ABPPV
	Means ± SD				*P*-value		
TUG time (sec)	11.58 ± 2.24	17.48 ± 7.74	11.73 ± 5.43	0.001*	0.001**	0.994	0.001**
TUG number of steps	14.68 ± 2.01	19.55 ± 6.11	15.91 ± 1.82	0.001*	0.001**	0.545	0.007**
Stride length (meter)	1.04 ± 0.20	1.04 ± 0.28	1.77 ± 0.45	0.001*	1.000	0.001**	0.001**
Stride time (sec)	1.08 ± 0.15	1.22 ± 0.39	1.11 ± 0.14	0.146	0.146	0.895	0.318
Stride velocity (m/sec)	0.97 ± 0.19	0.94 ± 0.37	1.62 ± 0.44	0.001*	0.963	0.001**	0.001**
Turn time (sec)	1.59 ± 0.55	3.14 ± 1.76	2.23 ± 0.61	0.001*	0.001**	0.159	0.024**
Turn number of steps	2.73 ± 0.77	4.27 ± 1.32	3.45 ± 0.80	0.001*	0.001**	0.047**	0.001**

* *p*<.05; One-way ANOVA, **p<.05 Post-Hoc Tukey’s, BPPV: Benign Paroxysmal Positioning Vertigo, OAWBPPV: older adults without BPPV, OABPPV: older adults with BPPV, ABPPV: adults with BPPV.

Older adults with BPPV took a significantly higher number of steps to complete the TUG test. than adults with BPPV and older adults without BPPV group: F(2, 63) = 9.452, *p* = 0.001. Post hoc analysis revealed that the older adults with BPPV took a significantly higher number of steps compared to older adults without BPPV and adults with BPPV (*p* = 0.001, respectively), while between the adults with BPPV and older adults without BPPV, no significant differences were found.

### Straight walking phase of TUG test

There was a statistically significant difference in stride length during the straight-walking phase of the TUG test among the three groups: F (2, 63) = 37.344, *p* = 0.001. The post hoc test revealed that the older adults with BPPV took shorter strides compared to adults with BPPV (*p* = 0.001) and adults with BPPV (*p* = 0.001). However, there were no significant differences in stride length during the straight walk phase of the TUG test between older adults without BPPV and older adults with BPPV (*p* = 0.963). For stride time, there was no significant difference between the three groups (F (2, 63) = 1.985, *p* = 0.146).

There was a statistically significant difference in stride velocity during the straight-walking phase of the TUG test among the three groups: F (2, 63) = 26.359, *p* = 0.001. Post hoc analysis revealed that the older adults with BPPV took a slower stride velocity compared to adults with BPPV (*p* = 0.001) and older adults without BPPV. However, there were no significant differences in stride velocity between older adults with BPPV and older adults without BPPV (*p* = 0.963).

### Turning phase of TUG

There was a significant difference in the time taken to complete the turn among the three groups: F(2, 63) = 10.5, *p* = 0.001. Post hoc comparisons revealed that the older adults with BPPV had a significantly longer turn time compared to older adults without BPPV and adults with BPPV (*p* = 0.001, 0.024), while between the adults with BPPV and older adults without BPPV it was not significant (*p* = 0.159).

There was a significant difference in the number of steps taken to complete the turn among the three groups: F(2, 63) = 13.33, *p* = 0.001. Post hoc comparisons revealed that the older adults with BPPV had a significantly longer turn time than older adults without BPPV and adults with BPPV (*p* = 0.001, respectively). Furthermore, adults with BPPV took a higher number of steps than older adults without BPPV (*p* = 0.047).

### Indicator of turning difficulty

Table 3 shows that none of the individuals staggered during turning. About 68.2% of healthy older adults, 95.5% of older adults with BPPV, and 90.9% of adults with BPPV experience no pivoting during turning. A higher percentage of older adults with BPPV accomplished turns in three seconds and more (50.0%) and used five or more steps during turns (31.8%) compared to other groups of participants. The older adults with BPPV have the highest turning difficulty indicators compared to the adults with BPPV and older adults without BPPV.

**Table 3.** Indicator of turning difficulty.

Category	OAWBPPV (*n* = 22)		OABPPV (*n* = 22)		ABPPV (*n* = 22)	
	Frequency	Percentage (%)	Frequency	Percentage (%)	Frequency	Percentage (%)
Presence of Staggering during Turning	0	00.0	0	00.0	0	00.0
Absence of pivoting during the turn	15	68.2	21	95.5	20	90.9
Accomplishes turn in 3.00 s or greater	1	4.5	11	50.0	1	4.5
The use of 5 or more steps/weight shifts to accomplish the turn.	0	00.0	7	31.8	2	9.1

BPPV: Benign Paroxysmal Positioning Vertigo, OAWBPPV: older adults without BPPV, OABPPV: older adults with BPPV, ABPPV: adults with BPPV.

## Discussion

To the best of our knowledge, this is the first study to explore gait changes during the straight walking and turning phase among individuals with BPPV. This study aims to determine the spatio-temporal gait parameters and indicators of turning difficulty during the TUG test compared to adults with BPPV and older adults without BPPV. We presented some important findings. Firstly, this study suggests that older adults with BPPV experience greater difficulty and impairment in mobility and balance, as evidenced by both the longer time taken and the higher number of steps required to complete the TUG test compared to the other groups. Secondly, older adults with BPPV exhibit altered gait parameters, specifically shorter stride length and slower stride velocity, during the straight-walking phase of the TUG test compared to their counterparts without BPPV. Third, older adults with BPPV face challenges in completing turns compared to their counterparts without BPPV. The prolonged turn time and increased number of steps taken by older adults with BPPV may reflect the impact of vestibular dysfunction on their mobility and balance. Additionally, we also found that older adults with BPPV presented with less pivoting during turning but spent longer and took more steps to perform the turning than the other 2 groups. There are differences in various gait parameters between older adults with and without BPPV, as well as disparities between older and younger adults, suggesting that aspects related to gait may be more influenced by age.

In general, the results showed that older adults with BPPV took longer time and a greater number of steps to complete the TUG test compared to older adults without BPPV. This may be due to a combination of factors, such as a more complex and challenging walking pattern caused by imbalance during walking and cautious movements to prevent falls and dizziness caused by head movements [27]. Head movements during walking can cause otoconia movement, which may aggravate dizziness and imbalance [1]. Therefore, older adults with BPPV may take cautious steps during the task to prevent falls. Older adults with BPPV may also require a stepping strategy to return to a more stable gait, which may provide a robust control approach to cope with altered sensorimotor function.

We found that older adults with BPPV have shorter stride length and slower stride velocity than older adults without BPPV. Previous studies have shown similar findings and attribute this to disturbances of sensory organization and abnormal vestibulospinal output caused by impaired vestibular information, leading to instability [2, 3, 28, 29]. Older adults with vestibular impairment tend to adopt a compensatory strategy, such as a shorter step, to improve dynamic stability during walking and prevent falls. Gait analysis of individuals with BPPV showed a decrease in the root mean square of the medial-lateral axis of both the head and the trunk, indicating a greater postural disturbance and a lower harmonic ratio indicating a lower smoothness of gait and poor postural control [2]. Lower gait quality was also assessed by significantly lower step regularity, stride regularity and gait symmetry, suggesting that individuals with BPPV were less able to regulate repeated walking pace and control a rhythmic displacement of the body during walking.

Older adults have the slowest stride velocity compared to other groups, as shown by their combination of the shortest stride length and longest stride time. This could be due to the strategy they choose when walking. In our study, older adults and those with BPPV tend to choose a self-selected walking pace during straight walking, which increases stability, consistent with a previous study [1]. The longer stride time may result from these individuals adapting their gait to optimize stability during walking. A decrease in vestibular input may also affect the dynamic stability of individuals with BPPV.

Dynamic balance control includes postural transition during changes in direction while walking, such as turning. Turning may be less automatic than straight walking because it requires complex dynamic balance control and sequential whole-body coordination [5–7]. This could be due to disruption of the vestibular system, which affects their ability to perceive movement and balance [28, 29]. Turning is a challenging task that relies heavily on maintaining a stable gaze and dynamic stability. Ineffective stimulation of the otoliths and semicircular canals limits the activation of the vestibular nerve and nucleus [3, 8, 30]. As a result, the signals sent to the muscles responsible for eye movement may not be effective, leading to difficulties turning. The proprioception system is more heavily during turning due to changes in the vestibular system and limited vision, resulting in a lack of stability. Therefore, older adults with BPPV take longer to optimize stability during turning as it has been established that deterioration of natural vestibular function deterioration occurs [9]. Taking additional steps may indicate that older adults with BPPV use a strategy to improve their stability while turning. This strategy involves taking more steps during the turning process, specifically, by turning the pivot. This type of turn is complex as it requires slowing down, rotating the body, and accelerating in a new direction [10]. However, this can pose a significant challenge for people with poor balance, particularly when transitioning from standing on both feet to standing on one foot and then back to standing on both feet.

This study also observed indicators of turning difficulty in older adults with BPPV. The three groups did not show any shaking during the turning task. Although Thigpen et al. [10], in a previous study, suggested that staggering during turning is an indicator of instability, the subjects in this study may have compensated for their loss of balance by slowing down their movements and taking smaller steps to accomplish turning. In the lower limb component, taking multiple steps was the most common pattern in older adults with BPPV. Using a multiple-step strategy instead of a pivot strategy may indicate a change in how these individuals walk, moving from a feedback mechanism to a feedback mechanism [14, 15]. The multiple-stepping turn is slower and requires more closed-loop movement, which seems to necessitate more feedback, whereas the pivot turn is a quick, open-loop movement that relies on a feedforward mechanism [10]. Due to the increased frequency of unsteadiness symptoms among older adults with BPPV, this condition may lead to altered turning methods. Older adults with BPPV may opt for the ‘steps’ strategy while turning to counteract unsteadiness and prevent falling. Studies have also linked turning difficulties with an increased likelihood of falling, as evidenced by increased steps taken and a loss of balance [31, 32]. Most older adults with BPPV showed changes in their ability to turn, which may indicate difficulty turning. Turning among older adults with BPPV is characterized by a lack of pivoting and use of steps, a longer time to complete turns (taking more than 3 min), and the use of multiple steps (5 or more) to complete the turn. Thus, they could use the turning steps to optimize their safety and reduce loss of balance and fear of falling.

The study may have limitations due to the lack of a detailed assessment of sarcopenia as a contributing factor. Sarcopenia, which is characterized by reduced muscle mass and function, has been linked to gait speed and overall physical performance in older adults. Future research in this area should aim to incorporate assessments of muscle mass, strength, and function to better understand the interplay between sarcopenia and BPPV-related gait abnormalities. Another study’s limitation is the absence of intra- and inter-reliability assessments of the results obtained from the randomized sample data analyzed by two researcher assistants. Future studies should aim to incorporate these reliability assessments as standard practice to strengthen the methodological rigor of the research.

## Conclusions

The findings of this study contribute to a deeper understanding of how BPPV influences walking in older adults. Examining gait changes in individuals with BPPV helps us understand the gait changes that occur in these individuals, despite the report of unsteadiness while walking. The main finding, that older adults with BPPV have difficulty turning, emphasizes the need to assess gait during turning and can be used to assess mobility and fall risk. Early recognition and identification of gait impairment can prevent falls and related health complications in people with BPPV.

## Data Availability

The data that support the findings of this study are available from the corresponding author, HM, upon reasonable request.
